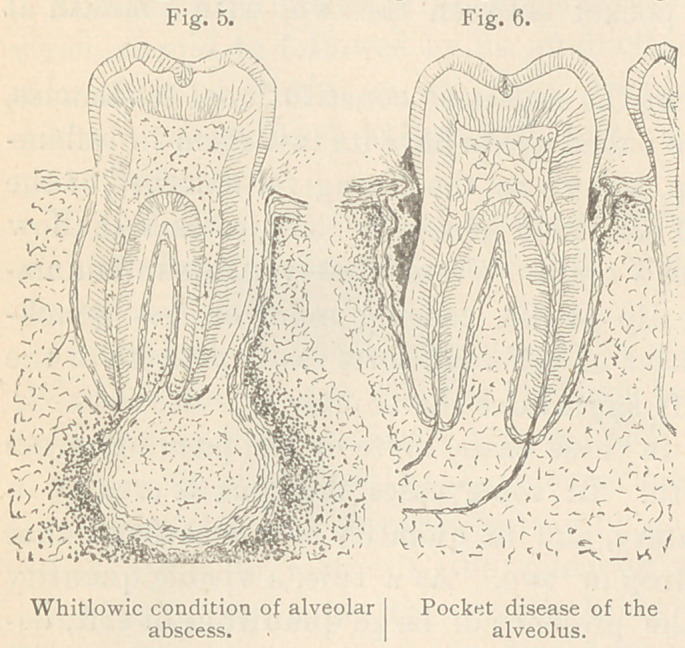# Pocket Disease of the Alveolus

**Published:** 1886-04

**Authors:** J. N. Farrar

**Affiliations:** New York City


### POCKET DISEASE OF THE ALVEOLUS.

BY J. N. FARRAR, M. D., D. D. S., NEW YORK CITY.

(Continued from page 513, Vol. VI.)

As injurious as the malady termed alveolar abscess may be to the usefulness of teeth, there is a disease of the socket known as "Pyorrhea Alveolaris," which in its consequences is more detrimental.

A disease belonging to middle and later life, it attacks at all ages after childhood. Although seldom found as early as at ten years of age, it is not infrequent at twenty, very common at forty, and (in some countries) almost universal at sixty.

The chief characteristic of this lesion, which in its unchecked course passes from bad to worse through several successive stages, is separation of the lining membrane of the socket (scientifically denominated pericementum) from portions of the root of the tooth, thus causing a pouch or pocket between the two, with a mouth at the margin of the gum.

Deferring for the present the aspect of constitutional tendencies, this pocket appears to the naked eye to have its initiation in inflammation of the annular lip, known as the "gingival margin" of the gum, which in its normal condition generally constitutes a shallow trough around the neck of a tooth. From whatever this inflammation arises, the ring-like lip swells, causing more or less pouching, so that irritating matter of different sorts easily collects in the trough, which increasingly aggravates the trouble.

Often, if not generally in these cases, more or less pus discharges at the necks of the teeth. In some cases this pus is creamy in appearance, in others watery, and in quantity it varies from that which is invisible to a drop or two. As a rule, a visible quantity of creamy pus indicates the presence of large quantities of soft, degenerated deposits in the pocket, or rough, sharp incrustations of earthy matter upon the root, while a watery discharge is an evidence that the deposit is scanty in quantity, or comparatively smooth. Cases belonging to the latter class are generally more or less chronic.

After this disease has become passive it requires but slight irritation to keep it along. In fact, nothing more seems necessary than the alteration of the juices that ooze from the blood through the walls into the pocket, which soon becomes rancid, and made worse by microbes, which are generally, if not always, present. These are the cases which are sometimes cited as proof that this disease may arise independently of local irritations. But upon this point more is intended at another time.

While entire sockets are occasionally affected, most of them are attacked only on one or two sides, ranging from a slight distance from within the annular lip to the entire length of the root.

In external appearance this pyorrhea (pus flow) resembles that which I denominated in a course of lectures delivered at Philadelphia in 1878,* a whitlowic condition of alveolar abscess; a condition where the pus from the sac, instead of finding vent by the usual route, through the wall of the socket, travels along between its lining membrane and the root and makes its exit at the neck of the tooth. But the two diseases differ widely in cause and effect, as well as in location of their incipient stages, as will be seen by comparing the following diagrams. Fig. 5 illustrates a sectional view of a perfected whitlowic alveolar abscess, in a pyorrhœic condition, discharging at the neck of a molar tooth.

Fig. 6 is a sectional view, illustrating pocket disease of the alveolus, with a deep pouch on one side, and a shallow one on the opposite of a molar tooth, both in a pyorrhœic condition, discharging at the neck, similar in external appearance to that shown by Fig. 5. Both of the pockets in Fig. 6 illustrate (in block) irritating calcareous deposits upon the roots.

Fig. 5.

Whitlowic condition of alveolar abscess.

Fig. 6.

Pocket disease of the alveolus.

Differentiately speaking, one of these diseases kindles comparatively sudden, and is generally violent in development; the other is slow and insidious. One begins in the interior of the jaw, around about the end of the root, in the form of a tumor, and generally results in an abscess; the other starts on the exterior, just within the gingival margin of the gum, and by a sort of ulcerative process works down between the lining membrane of the socket and the root. (Never "beginning about the end of the root," nor is the cause the "same in kind as that which leads to exostosis or enlargement of the root.") One seems to make an effort to rid the part of an evil irritant by delivering it from within outwardly, generally through the side of the socket, as if attempting to do as little harm to the tooth as possible; the other from without inwardly, as if

* Published in *Missouri Dental Journal*, 1879.

bent on doing all the mischief possible, first to the socket by the "wasting process," second by the loosening and final ejection of the tooth, soon after which the disease generally vanishes. In short, one seems to act on the defensive, the other on the offensive.

Those rare cases of so-called alveolar abscesses which are said to form on the side of the roots of teeth having living pulps, where there are no pockets opening at the cervical margins of the gums, are not of this variety of socket disease. There are, however, two conditions of abscess that are the result of detached sharp fragments of calcareous deposits from the roots caused by this disease, or in later stages of the pocket disease, after having effected a carious condition of the alveolar process, which, although bodily not dead tissue, yet from decomposition of minute particles here and there, or from stasis in the soft tissues in the rear of the walls of the pocket, evolve gas, etc., and cause tumefaction outside of the alveolar process at greater or less distance from the pocket, but as these must be considered more in the light of "sequelæ" they do not strictly belong to the present aspect of the question, hence will not be further dwelt upon at this time.

In order to prepare your mind, I will anticipate some features of my story by briefly stating that it has been taught, and the notion appears to be generally believed, that there is always necrosis of some portion of the alveolar process in these cases, and that without it a true type of the disease does not exist, and based upon this, excision of the hard tissus has become almost a universal treatment.

Although sometimes present in later stages of the disease, I am led to believe, from careful investigation and treatment upon an average of about fifty teeth per day for several years, that this disease must really exist long before the alveolus can possibly become carious, a condition which, in some form, must precede necrosis. Furthermore, I think that although more frequently present among that portion of the lower orders of society which do not get their teeth extracted whenever slightly annoying, caries and necrosis exist only in a small percentage of cases. Especially so among the middle and upper classes, who are more in the habit of caring for their teeth.

At this time this statement may seem like heresy, but it is based upon careful records of every case in my practice for ten years, which show that only about one per cent. of the patients (not the number of teeth) was afflicted with necrosis of the alveolus, the proof of which laid in the cure without surgically interfering with the alveolar process. The same evidence exists in the *almost* universally rapid cure after extraction, which would not be so if necrotic tissue remained.

Although my own practice shows only one per cent., I do not pretend that the experience of others must be the same; but supposing that others should find double the percentage, it would not materially change the basis of the conclusions. Although necrosis of the alveolar process is rare, the death and degeneracy of that portion of the cementum constituting one of the walls of the pockets is not common.

While this disease is as old as civilization, it received but little attention from the profession until a few years ago, and until of late was supposed to be incurable except by extraction of the tooth; but it is now known to be otherwise, notwithstanding it is pretended that all treatment is as yet empirical, on the ground that the etiology of the disease is not fully understood.

In such a state of things, as with all questions in dispute, various hypotheses and notions abound, some of which seem reasonable, others amusingly absurd, making it appear that all are groping in the shades of uncertainty.

Even the matter of a name for the disease is unsettled. While some people say that one name is as proper as another, and think that Tom Jones is a name for his characteristics, others think that in order to be as scientific and practicable as possible the characteristics of a disease should in a measure be expressed in the derivatives of the name. The term Pyorrhœa (pus flowing), although satisfactory to some people, with others is not; and although I am averse to innovations in nomenclature once accepted by any considerable number of people, I must say that with the dissatisfied I incline, for several reasons. 1st, the lesion does not always flow pus; 2d, it fails to express any idea of the chief and constant characteristic of the lesion (pocket); and, 3d, it fails to carry in its meaning sufficient diagnostic value to differentiate the disease from the other pus-flowing socket (whitlowic alveolar abscess), which also discharges at the neck of the tooth.

If what has been said be true, that the disease, so far as can be seen by the naked eye, commences about the neck of the tooth, and extends down the socket-causing pockets, and if true that pus is caused by irritation from deposits within, and the disease vanishes when the pockets are carefully and thoroughly cleaned, and *kept clean*, or when the teeth are extracted, and the main conditions of the lesion differ from that of a whitlowic condition of alveolar abscess, and if alveolar caries or necrosis, if present at all, belongs to later stages of the disease, and is a result rather than a cause, then the suggestion naturally presents itself: Ought not the general name to be fixed upon the most pronounced constant feature?

Supposing it were possible to find a term that would by some modification differentiate the two kinds of "pus flowing," would it be best to confine the meaning to simply the act of flowing of pus, when it is well known that the act in a large percentage of cases is not visible? Admitting this, for sake of the argument, would it be much better to confine its meaning to any one of the stages, which, if present at all, would, by its transitional acts, soon lose identity? Even if comparatively stationary, would it be well to fix upon stage conditions, such as when the surfaces of the walls of the pocket have become pyogenic, or when the alveolar process has become carious, or perhaps necrotic, when we know that any one, or all, may possibly be wanting? On the other hand, if the lesion has a peculiar characteristic in the pouch or pocket formation, and this is the only constant characteristic feature of the lesion, does it not have the strongest claim to the name? It seems so to me, and for that reason I generally use the term in preference to Magitot's.

While I generally prefer to use the English language in expressing the name, I sometimes use the Latin equivalent, Marsupiosis (pouch disease), or Loculosis (pocket disease), adding Alveolaris, which, rendered in full, is Marsupiosis Alveolaris, and Loculosis Alveolaris (pocket disease of the alveolus). Of the two terms, the latter probably would generally be considered the more euphonious.*

---

* Loc (us)=a place.

(ul)=diminutive suffix.

Locul (us)=a little place, or hole; a fold in the "toga," used as a pocket.

os (is)=a Greek termination denoting the becoming, or the change to a certain state; as, necrosis, "the becoming dead."

Loculosis=the formation of, or "becoming," a pocket; or a disease which consists in the formation of a pocket.

Alveolaris=pertaining to, or situated in, the alveolus.

Loculosis Alveolaris=pocket disease located in the alveolus. Pocket disease of the alveolus.

To distinguish one phase of the disease from another, characteristic expressions, or numerals in the natural order of the successive stages, may be used. Thus, inflammation of the gingival margin of the gum (gingivitis) may be known as the first stage, while a later one, when the pocket has become established, may be known as the second, the carious as the third, and the necrotic as the fourth stage.

When, in order to be explicit, it is desirable to express the recurrent form of this disease (after once cured), which is liable in cases where death or low vitality of the cemental wall of the pocket prevents reunion with the pericemental wall, the termination *osis* may be changed to *itis*, thus: Loculitis (disease *of* the pocket), which easily distinguishes it from the original pocket disease.

Another modification may be convenient—the changing of the terminations above mentioned to *ic*—thus, Loculitic (as in the expression, "The sockets are in a loculitic condition).

To reiterate, these brief, explicit and easily-spoken terms in a nutshell, are as follows:

Loculosis Alveolaris = Pocket disease of the Alveolus.
Loculitis = Disease of the pockets (recurrent form).
Loculitic = In the pocket disease condition.
First stage = Gingivitis.
Second stage = Pocket established.
Third stage = Carious condition of the alveolar process.
Fourth stage = Necrotic condition of the alveolar process.

## Figures and Tables

**Fig. 5. Fig. 6. f1:**